# Chronic Occupational Exposure to Ionizing Radiation Induces Alterations in the Structure and Metabolism of the Heart: A Proteomic Analysis of Human Formalin-Fixed Paraffin-Embedded (FFPE) Cardiac Tissue

**DOI:** 10.3390/ijms21186832

**Published:** 2020-09-17

**Authors:** Omid Azimzadeh, Tamara Azizova, Juliane Merl-Pham, Andreas Blutke, Maria Moseeva, Olga Zubkova, Natasa Anastasov, Annette Feuchtinger, Stefanie M. Hauck, Michael J. Atkinson, Soile Tapio

**Affiliations:** 1 Helmholtz Zentrum München—German Research Centre for Environmental Health GmbH, Institute of Radiation Biology, 85764 Neuherberg, Germany; natasa.anastasov@helmholtz-muenchen.de (N.A.); Atkinson@helmholtz-muenchen.de (M.J.A.); soile.tapio@helmholtz-muenchen.de (S.T.); 2 Southern Urals Biophysics Institute (SUBI), Russian Federation, 456780 Ozyorsk, Russia; clinic@subi.su (T.A.); moseeva.maria@mail.ru (M.M.); zubkova_subi@mail.ru (O.Z.); 3 Helmholtz Zentrum München—German Research Centre for Environmental Health, Research Unit Protein Science, 80939 Munich, Germany; juliane.merl@helmholtz-muenchen.de (J.M.-P.); hauck@helmholtz-muenchen.de (S.M.H.); 4 Helmholtz Zentrum München—German Research Centre for Environmental Health GmbH, Research Unit Analytical Pathology, 85764 Neuherberg, Germany; andreas.parzefall@helmholtz-muenchen.de (A.B.); annette.feuchtinger@helmholtz-muenchen.de (A.F.); 5 Chair of Radiation Biology, Technical University of Munich, 81675 Munich, Germany

**Keywords:** FFPE, ionizing radiation, occupational exposure, proteomics, label-free, PPAR alpha, heart, ischemia, cardiovascular disease

## Abstract

Epidemiological studies on workers employed at the Mayak plutonium enrichment plant have demonstrated an association between external gamma ray exposure and an elevated risk of ischemic heart disease (IHD). In a previous study using fresh-frozen post mortem samples of the cardiac left ventricle of Mayak workers and non-irradiated controls, we observed radiation-induced alterations in the heart proteome, mainly downregulation of mitochondrial and structural proteins. As the control group available at that time was younger than the irradiated group, we could not exclude age as a confounding factor. To address this issue, we have now expanded our study to investigate additional samples using archival formalin-fixed paraffin-embedded (FFPE) tissue. Importantly, the control group studied here is older than the occupationally exposed (>500 mGy) group. Label-free quantitative proteomics analysis showed that proteins involved in the lipid metabolism, sirtuin signaling, mitochondrial function, cytoskeletal organization, and antioxidant defense were the most affected. A histopathological analysis elucidated large foci of fibrotic tissue, myocardial lipomatosis and lymphocytic infiltrations in the irradiated samples. These data highlight the suitability of FFPE material for proteomics analysis. The study confirms the previous results emphasizing the role of adverse metabolic changes in the radiation-associated IHD. Most importantly, it excludes age at the time of death as a confounding factor.

## 1. Introduction

Epidemiological studies in the Mayak Production Association occupationally exposed to chronic low-dose-rate radiation have shown a significant increase in the incidence of ischemic heart disease (IHD) causally associated with the total external gamma-ray dose. The association remains after correction for multiple competing factors such as smoking and alcohol consumption [1,2,3,4].

Our previous proteomics study using fresh-frozen tissue obtained at autopsy from the cardiac left ventricle from Mayak workers showed a dose-dependent downregulation of proteins involved in mitochondrial energy metabolism, cardiac cytoskeleton, and oxidative stress response when compared to the samples from non-irradiated controls [5,6]. However, in this study, the individuals in the occupationally exposed group (>500 mGy) were on average 10 years older than the control subjects [5]. We applied a statistical method to distinguish the dose-only dependent protein expression changes from those of the age-only dependent changes. The analysis indicated that the majority of the proteins were deregulated by the radiation dose and not the age [6]. However, the confounding role of age could not be totally excluded. To address this issue, we initiated a new study where the control group was older than the irradiated group. Due to the difficulty of obtaining additional fresh-frozen material, we decided to use formalin-fixed, paraffin-embedded (FFPE) left ventricle tissue in the new study. 

FFPE tissue is an ideal source of analytical material for clinical investigations due to its stability in long-term storage [7,8,9,10,11,12,13]. The technical improvement of proteome analysis using FFPE material is a prerequisite for the successful elucidation of biological pathways. However, in spite of several technical improvements, proteomic analysis using FFPE material as an alternative to fresh-frozen tissue remains a challenge [14,15,16,17,18,19,20,21]. The harsh conditions typically used for fixation have previously prohibited successful protein identification and reproducible protein quantification [18,19,22,23]. During the formalin fixation proteins undergo degradation and cross-linking. The fixation results in a 30 Da Hydroxymethyl modification, tagged mainly on lysine residues contributing to the inter- and intramolecular cross-linking reactions [19,22,24,25]. Lysine modification affects the efficiency of protein extraction and digestion [19,26,27]. The Mass Spectrometry analysis often showed a general preferential detection of tryptic peptides with the C-terminal arginine over lysine indicating that modified lysine residues are inaccessible to protease during digestion [19,26,27].

A variety of methods that have been developed allowing the extraction of proteins from FFPE samples [9,28,29,30] have proven to be quite successful in performing proteomics analysis [9,31]. However, only a few proteomics studies using human FFPE cardiac tissue exist, probably due to the lack of this type of biomaterial [32,33]. 

The data presented here show the compatibility of FFPE tissue for a proteomic approach. The study suggests that chronic radiation exposure induces alterations in the heart metabolism, structure and oxidative stress response that may contribute to the radiation-induced IHD. Furthermore, it shows that these alterations are not caused by normal ageing.

## 2. Results

### 2.1. The Proteomics Analysis of FFPE Human Heart Tissue Provided a Comprehensive Cardiac Proteome Coverage

To investigate the effect of chronic radiation exposure on the cardiac left ventricle we compared the proteomes of FFPE samples collected from 5 control individuals not exposed to radiation with those from 15 individuals exposed to cumulative external gamma radiation doses of more than 500 mGy (Table S1). 

**Figure 1:**
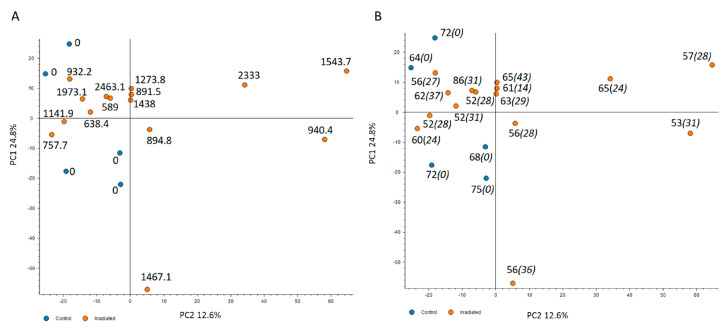
The principal component analysis (PCA) based on all proteomics features. The PCA used all features resulting in PC1 and PC2 as follows: PC1 24.8% and PC2 12.6%. The distribution of proteomics features is illustrated based on the dose (mGy) (**A**), the age (regular font) and the years of exposure (italics) (**B**). The control samples are presented as blue spots and the irradiated samples with the corresponding individual dose in orange. The analysis was performed using the Proteome Discoverer 2.3 software (Thermo Scientific, Waltham, MA, USA). Detailed information of the sample donors and the exact doses are given in Table S1.

The changes in the cardiac proteome were analyzed with label-free quantitative proteomics. A principal component analysis (PCA) using all proteomics features revealed that the irradiated samples were to some extent but not dose-dependently separated from the controls (Figure 1A). To examine whether the age of the individuals or the time of exposure may affect the distribution of proteome features, these two factors were merged on the PCA (Figure 1B and Table S1). The analysis confirmed that neither the age nor the years of exposure affected the distribution between the two groups (Figure 1B).

**Figure 2:**
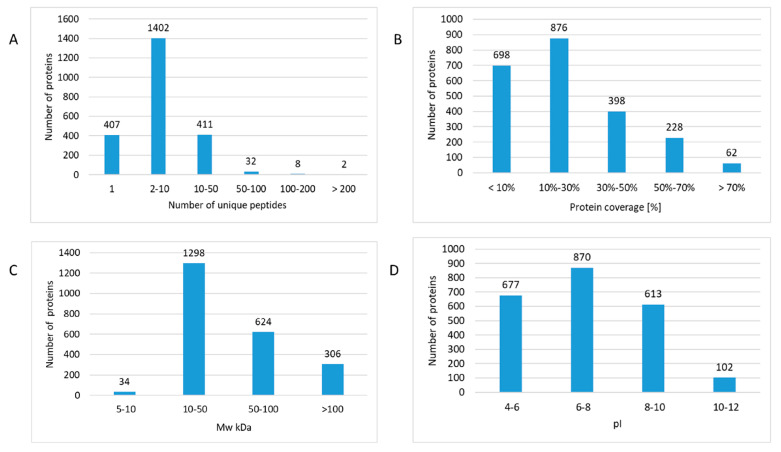
The features of protein identifications in formalin-fixed paraffin-embedded (FFPE) proteomics. The number of identified proteins was assigned based on the number of unique peptides (**A**), protein coverage (**B**), molecular weight (MW kDa) (**C**), and isoelectric point (pI) (**D**). The analysis was performed using the Proteome Discoverer 2.3 software (Thermo Scientific, Waltham, MA, USA). Detailed information of the proteomics features is given in Table S2.

A total number of 2663 proteins were identified and quantified, of which 1855 proteins were quantified with at least two unique peptides across multiple samples (Table S2). The majority of proteins were identified with 2–10 unique peptides (Figure 2A and Table S2). Surprisingly, this indicated markedly more identifications than in our previous study using fresh-frozen material with 1281 identified proteins [5]. 

Furthermore, a broad dynamic range of cardiac proteome was covered since almost 70% of all proteins were identified with sequence coverages up to 30% (Figure 2B and Table S2). The theoretical molecular weight of the identified proteins ranged from 5 kDa (thymosin beta-10) up to 3814 kDa (titin). The molecular weight of the majority (57%) of the identified proteins was between 10–50 kDa but also several high molecular weight (>100 kDa) proteins were identified (Figure 2C and Table S2). The analysis covered a broad range of cardiac proteins with different isoelectric points (pIs) (Figure 2D and Table S2). The pI values ranged from 3.78 (prothymosin alpha) to 12.56 (60S ribosomal protein L39) (Table S2). Strongly basic proteins including different mitochondrial subunits and ribosomal proteins were identified (Table S2).

In order to investigate the efficiency of protein extraction to revert the cross-linking modifications, a 30 Da hydroxymethyl modification was set for the lysine and arginine during searching against the database [19]. The reanalysis of the generated 28,798 peptides revealed that only 2.7% of all identified peptides contained modified lysine or arginine residues (Table S3) suggesting an efficient removal of modifications induced by cross-linking during protein extraction. 

**Figure 3:**
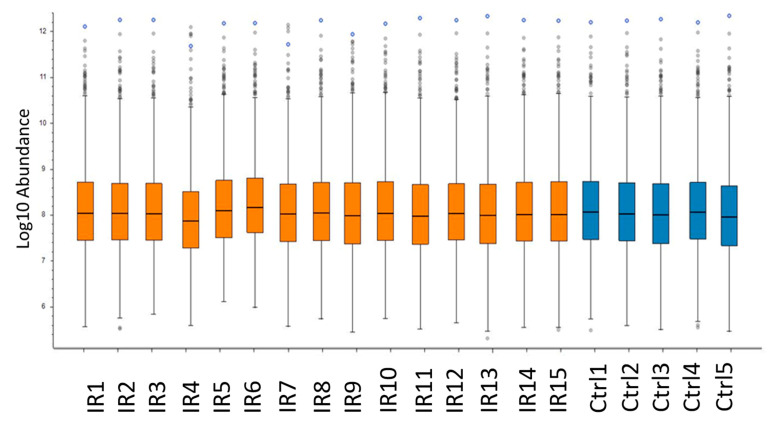
Comparison of protein abundance between heart samples. The box-and-whisker plot shows the abundance of the not-normalized intensity values for each individual of irradiated (IR) and control (Ctrl) groups. The analysis was performed using the Proteome Discoverer 2.3 software (Thermo Scientific, Waltham, MA, USA). Detailed information of the sample donors and the exact doses are given in Table S4.

To investigate the effect of the heterogeneity of the individuals on the variance of the proteome profiles between samples, the abundance of the non-normalized intensity values for each individual was compared (Figure 3 and Table S2). Despite the expected heterogeneity, the analysis showed a uniform distribution for the intensity of identified proteins between individuals. This finding was in a good agreement with the coefficient of variation (CV) of the protein intensity detected in different individuals. The mean values of CV% were 13.10 and 13.83 for protein abundances in irradiated and control groups, respectively (Table S4).

### 2.2. Proteomic Analysis of FFPE Cardiac Tissue Showed Alteration of the Heart Proteome after Chronic Irradiation

Among the quantified proteins with two or more unique peptides, the expression level of 196 proteins was significantly different between irradiated samples and controls (2 unique peptides; ±1.3-fold; *p* < 0.05). Of these, 105 proteins were downregulated and 91 upregulated in the irradiated samples (Table 1). 

**Figure 4:**
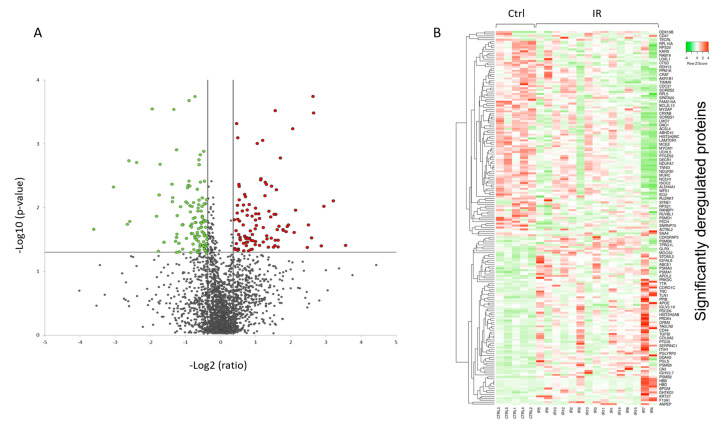
Graphical representation of quantitative proteomics data of human FFPE cardiac tissue after chronic irradiation. Proteins are ranked in a volcano plot according to the −log10 of their statistical *p*-value (y-axis) and log2 fold change (x-axis). The green and red points represent the significantly downregulated and upregulated proteins, respectively (**A**). The heat map shows hierarchical clustering (average linkage, Spearman ranked correlation) of significantly deregulated proteins in 5 controls (Ctrl) and 15 irradiated (IR) samples (**B**). The green bars indicate downregulation and the red bars upregulation. The analysis was performed using Heatmapper web server [34]. Detailed information of the proteomics features and individuals is given in Tables S1 and S2.

A volcano plot of all quantified and significantly deregulated proteins is shown in Figure 4A. The heat map based on the intensity of differentially regulated proteins in all individual samples showed a clear difference between the control and irradiated groups (Figure 4B).

Among the differentially regulated proteins were several lipid metabolism enzymes (Table 1). Long-chain-fatty-acid—CoA ligase 4 (ACSL4), hydroxyacyl-coenzyme A dehydrogenase (HADH), 2,4-dienoyl-CoA reductase (DECR1), enoyl-CoA hydratase (ECHS1), isovaleryl-CoA dehydrogenase (IVD), and enoyl-CoA delta isomerase 2 (ECI2) were all downregulated.

A number of proteins belonging to the sirtuin pathway and mitochondrial respiratory chain were also significantly downregulated in the irradiated samples (Table 1). The level of mitochondrial import inner membrane translocase (TIMM9), mitochondrial import receptor (TOMM22), and that of several proteins in the mitochondrial complexes I and V was reduced in the irradiated samples (Table 1).

Similarly, the data also showed downregulation in the expression of actin cytoskeleton components such as troponin I (TNNI3), troponin T (TNNT2), and different myosin isoforms including myosin heavy chain 14 (MYH14), myosin regulatory light chain 2 (MYL2), myosin light chain 4 (MYL4), and myomesin-1 (MYOM1) (Table 1). In contrast, several collagen isoforms were upregulated in the proteomics data, including collagen type XII alpha 1 chain (COL12A1), collagen type XV alpha 1 chain (COL15A), collagen type VI alpha 1 chain (COL6A) and collagen type VI alpha 2 chain (COL6A2) (Table 1).

The levels of several proteins of the oxidative stress response, namely catalase (CAT), peroxiredoxin 2 (PRDX2), peroxiredoxin 4 (PRDX4), extracellular superoxide dismutase [Cu-Zn] (SOD3), glutathione hydrolase 5 (GGT5), and glutaredoxin-1 (GLRX), were altered, most showing upregulation (Table 1).

**Figure 5:**
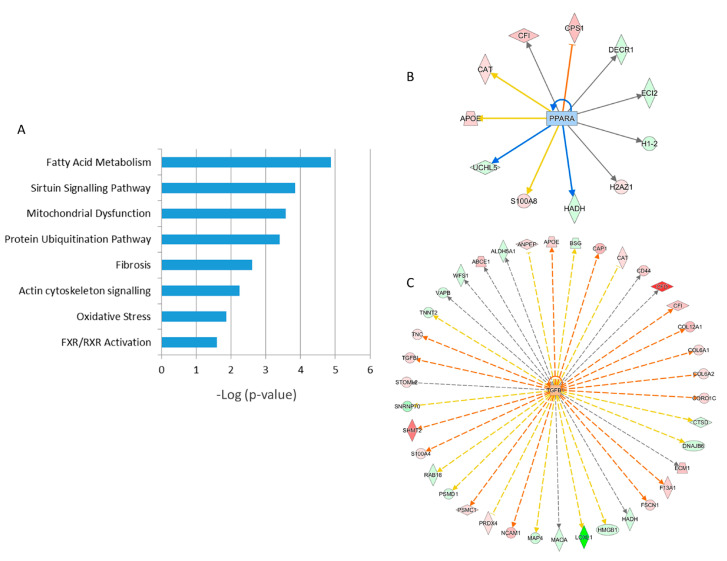
Pathway analysis of differentially regulated proteins from human FFPE heart tissues. The most significant canonical pathways altered by irradiation are shown. The analyses were generated using Ingenuity Pathway Analysis (IPA) (QIAGEN Inc., Hilden, Germany, https://www.qiagenbio-informatics.com/products/ingenuity-pathway-analysis) (Table S5). The bars indicate canonical pathways and the y-axis displays the −(log *p*) enrichment significance. The tall bars are more significant than the short ones (**A**). Graphical representation of the deregulated protein networks with their upstream transcriptional regulators peroxisome proliferator-activated receptor (PPAR) alpha (**B**) and transforming growth factor beta (TGFB) (**C**) are shown. The upregulated proteins are marked in red and the downregulated in green. The nodes in blue (inhibition) and orange (activation) represent transcription factors. The full protein names are given in Table 1.

A detailed analysis of the functional interactions and biological pathways of the deregulated proteins was performed using Ingenuity Pathway Analysis (IPA) software. Fatty acid metabolism, sirtuin signaling, mitochondrial dysfunction, protein ubiquitination, tissue fibrosis, cytoskeleton organization, and oxidative stress were the most affected pathways in the irradiated samples compared to the control (Figure 5A and Table S5). Especially two biological networks showed enrichment of deregulated proteins. The predicted regulators of these networks were the peroxisome proliferator-activated receptor (PPAR) alpha and transforming growth factor beta-1 (TGFB1) (Figure 5B,C, Table S5). PPAR alpha was predicted to be inactivated in irradiated samples, whilst TGF beta was predicted to be activated. Differentially expressed proteins were associated with cardiotoxicity-related pathways including cardiac dilation, cardiac enlargement, cardiac dysfunction, heart failure, and cardiac fibrosis (Table S5).

### 2.3. Chronic Irradiation Alters Myocardial Histomorphology

The proteomics data showed that the expression of several proteins belonging to the TGF beta signaling pathway was significantly changed in the irradiated hearts. These proteins included collagen isoforms that were all upregulated in irradiated samples. Therefore, we analyzed the deposition of collagen in FFPE cardiac tissue samples using a histomorphological approach (Figure S1A–C). 

The analysis displayed moderate to marked variation in the tissue composition regarding the proportions of the myocardium, epicardium, endocardium, coronary vasculature, and epicardial adipose tissue present on the respective sections, and presumable tissue locations of origin (ventricular or interventricular septal myocardium) between different individual samples of both groups. Similarly, the degrees and severities of histopathological alterations (fibrosis, inflammatory infiltrates, lipomatosis) varied between individuals.

**Figure 6:**
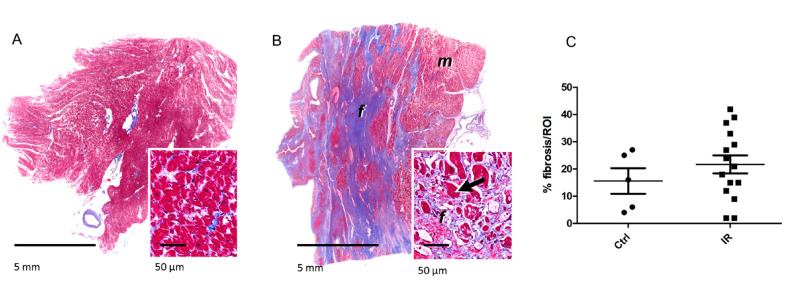
The detection of fibrosis in the heart samples. The sections were stained with Mason’s Trichrome for visualization of connective tissue. The fibrotic area was shown in representative sections of the myocardium in control (**A**) and irradiated samples (**B**). Myocytes (*m*) are stained in red, fibrous connective tissue (*f*) in blue. The arrows indicate degenerating cardiomyocytes entrapped by fibrosis. The areas of fibrotic tissue within the myocardium were quantified and presented as percentage of region of interest (ROI) (**C**). The icons represent individual samples in control (Ctrl) and irradiated (IR) groups. The details are given in Table S6.

Interestingly, the histopathological evaluation of tissue sections taken from the irradiated group revealed multifocal, moderate to severe interstitial myocardial fibrosis (Figure 6A,B). This was evident as large areas of coalescing foci of fibrotic or scar tissue extending into the myocardium. These foci replaced and surrounded individual cardiomyocytes and clusters of heart muscle cells. Additionally, moderate myocardial lipomatosis and multifocal interstitial lymphocytic and plasmacytic infiltrations were occasionally present in the irradiated samples. Cardiac vessels, particularly larger arteries displayed thickened vascular walls with expanded intimal and medial layers. The individual sections assigned to the control group also displayed variable histopathological lesions, including mild to moderate interstitial myocardial fibrosis and subacute severe myocardial degeneration and necrosis with vascular congestion and oedema, whereas two sections showed no relevant histopathological lesions (Figure 6A,B, and Table S6). On the average, the relative area of fibrotic tissue within the myocardium was 16 ± 11% in the control sections and 22 ± 13% in radiation-exposed heart tissue sections (Figure 6C and Figure S1C, and Table S6). Although the fibrotic area was bigger in most irradiated samples compared to the controls, this difference did not reach statistical significance.

### 2.4. Radiation-Induced Changes Identified by FFPE Proteome Profiling are Comparable to Those Observed in Irradiated Fresh Frozen Hearts

**Figure 7:**
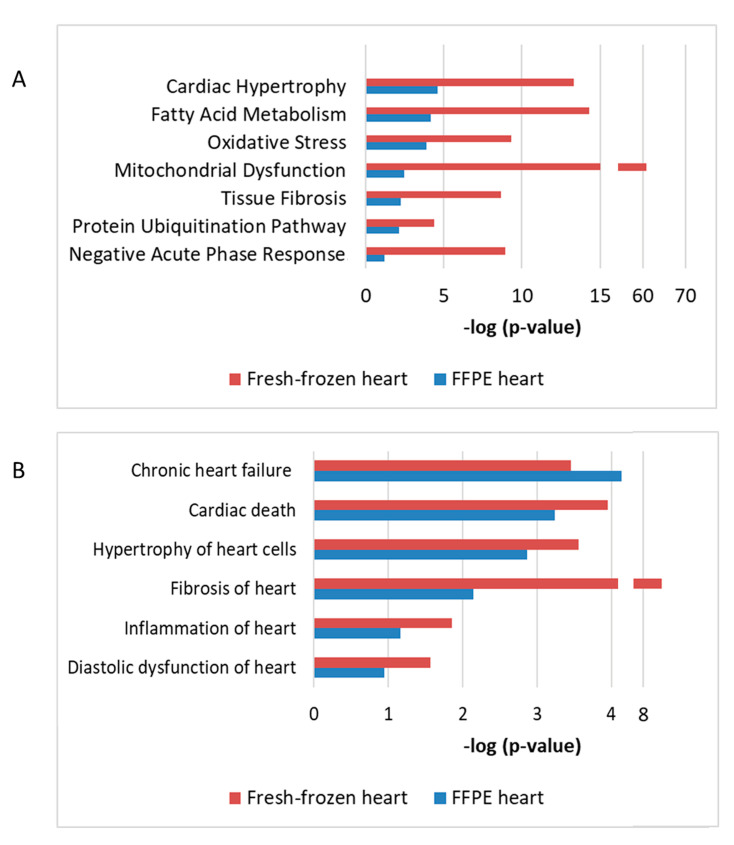
The comparison of proteomics data using FFPE or fresh-frozen cardiac material The enriched protein clusters of different affected pathways (**A**) and cardiac pathologies (**B**) were compared using data from the FFPE and fresh frozen cardiac tissue. The analyses were performed using IPA (QIAGEN Inc., Hilden, Germany, https://www.qiagenbio-informatics.com/products/ingenuity-pathway-analysis). The bars indicate affected pathways (**A**) or predicted diseases (**B**) and the y-axis displays the −(log *p*) enrichment significance. The tall bars are more significant than the short ones.

Using a protein–protein interaction and pathway analysis, we compared the proteomics data from the FFPE and previously analyzed fresh-frozen tissue [5]. Proteome profiling of both studies indicated similar cardiac response to chronic radiation exposure (Figure 7). A comparison between the studies indicated that chronic irradiation strongly affected cardiac metabolism, structure, and stress response (Figure 7A). Moreover, both analyses showed alterations in several proteins associated with heart pathologies including heart failure, fibrosis, inflammation, and hypertrophy (Figure 7B).

## 3. Discussion

This is, to the best of our knowledge, the first study performed on human FFPE heart tissue to analyze the late effects of chronic radiation exposure. The data presented here emphasize the suitability of FFPE material for proteomic investigation. The sequential urea/SDS extraction and filter-aided sample preparation (FASP) digestion have markedly improved the number of identified and quantified proteins in this kind of material [16]. Proteome profile of the FFPE material showed even more protein identifications than the one using fresh-frozen cardiac tissue [5]. However, a direct comparison of the two studies is difficult due to the differences in the experimental design, sample size, analytical performances in mass spectrometry, and applications used for quantification and data analysis. Furthermore, the controls and the workers included in the two studies were different individuals with a differing age distribution. Nevertheless, the main results of the two studies were similar both showing considerable radiation-induced changes in proteins involved in the heart metabolism and structure. Since the control group in this study was on the average older than the irradiated group, these changes were not resulting from ageing.

The downregulation of mitochondrial proteins in respiratory chain complexes was observed in both studies. This is also in agreement with our previous data from mice where local heart irradiation was shown to induce persistent functional and proteome alterations in cardiac mitochondria [16,35,36,37]. This was associated with reduced activity of the complex I [38]. 

In consonance with the previous human study [5], the proteomics data here indicated a cluster of oxidative stress response proteins. Mitochondrial dysfunction is accompanied by enhanced reactive oxygen species (ROS) production [39]. Persistently increased mitochondrial ROS level has been shown to result from local heart irradiation in mice [36,38]. 

The sirtuin signaling was one of the most affected pathways in the present study. Deficiencies in mitochondrial oxidative phosphorylation, in particular in complex I, lead to a decrease in NAD^+^/NADH ratio and disruption of sirtuin signaling in the heart [39]. We have recently shown decreased SIRT1 and SIRT3 activity associated with hyperacetylation of cardiac mitochondrial proteins following chronic irradiation in ApoE^-/-^ mice [40]. 

The proteomic analysis showed alterations in several lipid metabolism enzymes in irradiated hearts. The functional analysis of deregulated proteins analysis predicted inactivation of PPAR alpha, a key transcriptional regulator of fatty acid metabolism, in irradiated samples. The impairment of the PPAR alpha pathway and inhibition of lipid metabolism enzymes have been repeatedly reported in radiation-induced cardiac damage [5,37,41,42]. We have shown previously the upregulation of the inactive (phosphorylated) form of PPAR alpha in cardiac samples of Mayak workers [5]. 

Cardiac fibrosis is a well-known side consequence of radiotherapy [43,44]. In this study, we investigated the effects of chronic low-dose-rate radiation with cumulative doses much lower than used in therapeutic applications. Yet, the proteomics data indicated upregulation of collagen isoforms and predicted activation of TGF beta signaling in irradiated hearts. The morphology analysis also indicated large foci of fibrotic tissue in the irradiated samples although in comparison with the controls this did not reach statistical significance. Increased expression of proteins involved in the fibroblast to myofibroblast differentiation was shown in mice after high doses of local heart irradiation, with the activation of TGF beta playing a central role [41]. Increased concentration of TGF beta has been reported in the serum of Mayak workers exposed to external gamma rays suggesting a systemic elevation in the level of this cytokine in this cohort [45].

Increased collagen deposition and fibrosis may induce changes in actin cytoskeleton [46]. Myofibrillar remodeling is typically found in cardiac conditions affected by IHD [47,48] and seems to be present in the radiation-induced form of the disease as well as indicated even previously [5,42].

Taken together, this study gives support to the earlier findings suggesting that cardiac metabolic and structural impairment observed in radiation-induced IHD are two sides of the same coin [49]. The deregulation of structural proteins may well be coupled to mitochondrial damage, resulting in impairment in the energy supply and increased mitochondrial ROS production. Since the inactivation of PPAR alpha seems to play an important role in this vicious circle one possible way forward would be to test the impact of PPAR alpha agonists in preventing radiation-associated heart disease.

## 4. Material and Methods

### 4.1. Human FFPE Heart Samples

Biological samples were collected post mortem from donors who had previously given informed consent to participate in the study and who had consented to the processing of their data following the Russian Federal Laws № 323-FL of 27.09.2013 and № 261-FL of 25.07.2011. The study was approved by the Southern Urals Biophysics Institute’s Institutional Review Board and the formal approval No. 01/2011 as of 15.02.2011 was issued. The probands were male Mayak plutonium enrichment plant workers who were exposed only to external gamma rays. The control subjects were non-Mayak workers living in the same local area. All participants were diagnosed multiple times with IHD during their lifetime, and this was also listed as the primary cause of death. Workers exposed to internal plutonium (Pu alpha-activity in urine >0.5 kBq) or diagnosed with cancer or other major somatic diseases were excluded from the study.

The histology slides were prepared according to the conventional method used in hospital dissecting rooms and departments of morbid anatomy at research institutions of the Soviet Union and later the Russian Federation. Tissue resections were taken by an autopsist immediately during the autopsy procedure. A piece of the cardiac left ventricle (size 1 cm × 1.5 cm, 5 mm thick) was dissected from each organ and placed in a labelled cassette. Cassettes were put in containers with 10% buffered formalin fixative (pH between 7.2 and 7.4) for 24 h to 48 h. The volume of fixative was at least 20 times of the volume of the tissue specimen to ensure its complete infiltration with the fixative. After fixation, sample pieces were washed in the running tap water for 24 h to 36 h to remove redundant fixative. The older samples (before the year 2000) were serially dehydrated in an ethanol series from 50% to 100% and cleared in a mixture of absolute ethanol and chloroform to a final concentration of pure chloroform. Following dehydration and clearing of specimens, the samples were infiltrated and embedded using chloroform-paraffin mix and paraffin with beeswax. After the year 2000, xylene was used instead of chloroform for clearing. Then, the samples were infiltrated gradually in 2 containers of xylene and embedded in homogenized paraffin containing plasticizing agents with a melting temperature of 55 °C. Once the paraffin casing had solidified, blocks were taken out of the casts, sectioned with a microtome and stored in a repository.

For the current study, we used the FFPE heart tissues from 20 individuals, allocated between two dose groups as follows: 5 non-irradiated individuals to the control group (0 Gy), 15 Mayak workers to the dose group >500 mGy (Table S1). The smoking status and index, alcohol consumption and body mass index of each individual are indicated in Table S1. The individuals in the irradiated group were exposed to the radiation dose in range of 580 mGy to 2463 mGy (Table S1). The average age of the controls and irradiated group was 70 and 59.7 years, respectively (Table S1). Only one of the individuals from the irradiated group was older than 70 years (Table S1). 

### 4.2. Proteomic Sample Preparation and LC-MSMS Measurement 

FFPE tissue sections were deparaffinized by incubating twice with xylene for 10 min at room temperature before rehydration in a graded series of ethanol (100%, 95%, and 70%). De-crosslinking of FFPE tissue samples was performed in TBS for 60 min at 99 °C. After dilution with equal volumes of urea buffer (8 M urea in 0.1 M Tris/HCl pH 8.5), the tissue was lysed by homogenization in a Precellys homogenizer (Bertin, Montigny-le-Bretonneux, France). SDS was added to a final concentration of 0.2% before further incubation for 30 min at room temperature. Protein concentration was determined by Bradford assay. The lysed tissue was subjected to tryptic protein digest using a modified filter-aided sample preparation (FASP) protocol [21].

Equal peptide amounts per samples were measured on a Q-Exactive HF-X mass spectrometer (Thermo Scientific, Waltham, MA, USA) online coupled to an Ultimate 3000 nano-RSLC (Thermo Scientific, Dionex, Waltham, MA, USA). Tryptic peptides were automatically loaded on a C18 trap column (300 µm inner diameter × 5 mm, Acclaim PepMap100 C18, 5 µm, 100 Å, Thermo Scientific, Waltham, MA, USA) prior to C18 reversed-phase chromatography on the analytical column (nanoEase MZ HSS T3 Column, 100Å, 1.8 µm, 75 µm × 250 mm, Waters, Rydalmere, NSW, Australia) at 250 nL/min flow rate in a 95 min non-linear acetonitrile gradient from 3 to 40% in 0.1% formic acid. Profile precursor spectra from 300 to 1500 m/z were recorded at 60,000 resolution with automatic gain control target of 3e6 and maximum injection time of 30 ms. Subsequently, TOP15 fragment spectra of charges 2 to 7 were recorded at 15,000 resolution with an AGC target of 1e5, a maximum injection time of 50 ms, an isolation window of 1.6 m/z, normalized collision energy of 28 and a dynamic exclusion of 30 s.

The individual raw-files were loaded to the Proteome Discoverer 2.3 software (Thermo Scientific, Waltham, MA, USA) allowing for peptide identification and label-free quantification using the Minora node. Searches were performed using Sequest HT as a search engine in the Swiss-Prot human database with the following search settings: 10 ppm precursor tolerance, 0.02 Da fragment tolerance, two missed cleavages allowed, carbamidomethyl on cysteine as a fixed modification, deamidation of glutamine and asparagine and hydroxymethyl modification of lysine and arginine were allowed as variable modifications, as well as oxidation of methionine and Met-loss combined with acetylation at the N-terminus of the protein. After filtering for a 5% peptide and protein false discovery rate, proteins were quantified by summing up abundances of allocated unique and razor peptides. Resulting normalized protein abundances were used for calculation of fold-change and *p*-values of the individual proteins. Principal components analysis was exported from the Proteome Discoverer 2.3 software. For final quantifications, proteins identified with at least 2 unique peptides and with fold change ratios greater than 1.30-fold or less than 0.77-fold (*p* < 0.05) were defined as being significantly differentially expressed. Hierarchical clustering was made by Heatmapper online web server [34].

### 4.3. Protein–Protein Interaction and Signaling Network

The analyses of protein-protein interaction and signaling networks were performed by the software tool INGENUITY Pathway Analysis (IPA) (QIAGEInc., Hilden, Germany https://www.qiagenbioinformatics.com/products/ingenuity-pathway-analysis) [50]. 

### 4.4. Histopathology and Morphometric Analyses of Tissue Composition and Cardiac Fibrosis

Histopathological analyses were performed on 3 µm hematoxylin and eosin (HE)-stained paraffin sections. Masson’s Trichrome staining for connective tissue was used to demonstrate fibrosis. Histological sections were examined in a blinded fashion. The section areas of fibrotic tissue within the myocardium were quantified by digital image analysis using automated digital image analysis (Definiens Developer XD 2, Definiens^®^ AG, Munich, Germany). Epicardial tissue and heart valve section profiles were excluded from the analysis.

### 4.5. Statistical Analysis

Comparative analysis of the data was carried out using the Student’s *t*-test (unpaired). The significance levels were p* < 0.05. 

### 4.6. Data Availability

The raw MS data have been deposited in the RBstore database, Study ID: 1158: (https://www.storedb.org/store_v3/study.jsp?studyId=1158).

## 5. Conclusions

The data presented here show the applicability of the FFPE cardiac tissue in retrospective proteomic analysis with good identification of proteins of different molecular weight and pI. This is of advantage since attaining human biomaterial, especially cardiac material, for scientific purposes is in general complicated. Thus, using archival FFPE material now enables the investigation of molecular alterations associated with severe heart pathologies. This study demonstrates a series of radiation-induced changes in signaling pathways mainly involved in metabolic aspects probably contributing to this particular form of IHD. These data also exclude the confounding effect of the age in the radiation response of the heart.

## Figures and Tables

**Table 1:** Significantly deregulated proteins in irradiated cardiac left ventricle proteome. The proteins listed in the table were identified and quantified by two or more unique peptides; fold changes and *p*-values are shown.

Accession	ID	Annotation	Fold Changes	*p*-Value
P00746	CFD	Complement factor D	9.3	7.86 × 10^−3^
Q96PD5	PGLYRP2	N-acetylmuramoyl-L-alanine amidase	7.7	9.62 × 10^−3^
P04745	AMY1B	Amylase, alpha 1B	7.3	4 × 10^−2^
Q96JB5	CDK5RAP3	CDK5 regulatory subunit-associated protein 3	6.3	3.31 × 10^−4^
P34897	SHMT2	Serine hydroxymethyltransferase, mitochondrial	6.0	2.99 × 10^−2^
Q9BQE5	APOL2	Apolipoprotein L2	5.8	3.82 × 10^−5^
P20700	LMNB1	Lamin-B1	5.7	1.87 × 10^−2^
P05452	CLEC3B	Tetranectin	5.6	4.23 × 10^−2^
O60784	TOM1	Target of Myb protein 1	4.4	1.10 × 10^−2^
Q6ICL3	TANGO2	Transport and golgi organization 2 homolog	3.7	2.41 × 10^−2^
Q08209	PPP3CA	Serine/threonine-protein phosphatase 2B catalytic alpha	3.4	2.22 × 10^−2^
P37802	TAGLN2	Transgelin-2	3.3	1.67 × 10^−3^
Q01518	CAP1	Adenylyl cyclase-associated protein 1	3.3	1.84 × 10^−5^
A0A0A0MS15	IGHV3-49	Immunoglobulin heavy variable 3-49	3.2	1.98 × 10^−2^
P13591	NCAM1	Neural cell adhesion molecule 1	3.1	4.16 × 10^−2^
O95336	PGLS	6-phosphogluconolactonase	3.1	3.25 × 10^−2^
Q99715	COL12A1	Collagen alpha-1(XII) chain	3.1	2.22 × 10^−2^
O95865	DDAH2	N(G),N(G)-dimethylarginine dimethylaminohydrolase 2	3.0	5.28 × 10^−3^
Q5T0D9	TPRG1L	Tumor protein p63 regulated 1 like	3.0	3.05 × 10^−4^
P31327	CPS1	Carbamoyl-phosphate synthase, mitochondrial	2.9	3.27 × 10^−2^
P07738	BPGM	Bisphosphoglycerate mutase	2.9	1.28 × 10^−2^
P61221	ABCE1	ATP-binding cassette sub-family E member 1	2.9	4.21 × 10^−2^
Q7Z3Y8	KRT27	Keratin, type I cytoskeletal 27	2.7	4.64 × 10^−3^
Q16610	ECM1	Extracellular matrix protein 1	2.7	3.03 × 10^−2^
P05156	CFI	Complement factor I	2.7	1.41 × 10^−2^
P01780	IGHV3-7	Immunoglobulin heavy variable 3-7	2.6	1.14 × 10^−2^
P05546	SERPIND1	Heparin cofactor 2	2.6	5.00 × 10^−2^
O75122	CLASP2	CLIP-associating protein 2	2.6	4.21 × 10^−2^
Q2TAA2	IAH1	Isoamyl acetate-hydrolyzing esterase 1 homolog	2.5	3.13 × 10^−2^
P16070	CD44	CD44 antigen	2.4	4.01 × 10^−3^
P49721	PSMB2	Proteasome subunit beta type-2	2.4	6.87 × 10^−7^
P13667	PDIA4	Protein disulfide-isomerase A4	2.4	6.55 × 10^−3^
P35858	IGFALS	Insulin-like growth factor-binding protein complex acid labile	2.3	3.50 × 10^−2^
P19652	ORM2	Alpha-1-acid glycoprotein 2	2.3	2.06 × 10^−2^
O96007	MOCS2	Molybdopterin synthase catalytic subunit	2.3	8.91 × 10^−4^
P08294	SOD3	Extracellular superoxide dismutase [Cu-Zn]	2.2	5.01 × 10^−2^
Q6FI13	HIST2H2AA	Histone cluster 2 H2A family member a4	2.2	3.55 × 10^−3^
P00488	F13A1	Coagulation factor XIII A chain	2.2	3.71 × 10^−3^
P02649	APOE	Apolipoprotein E	2.2	3.95 × 10^−2^
Q9HBI1	PARVB	Beta-parvin	2.2	1.49 × 10^−2^
Q9BR76	CORO1B	Coronin-1B	2.1	1.26 × 10^−2^
P28074	PSMB5	Proteasome subunit beta type-5	2.1	9.94 × 10^−4^
P01714	IGLV3-19	Immunoglobulin lambda variable 3-19	2.0	1.91 × 10^−2^
O60888	CUTA	Protein CutA	2.0	1.25 × 10^−2^
Q16658	FSCN1	Fascin	2.0	3.42 × 10^−2^
Q9ULV4	CORO1C	Coronin-1C	2.0	1.02 × 10^−2^
P17655	CAPN2	Calpain-2 catalytic subunit	2.0	1.58 × 10^−2^
Q15582	TGFBI	Transforming growth factor-beta-induced protein ig-h3	2.0	3.83 × 10^−2^
P15144	ANPEP	Aminopeptidase N	1.9	3.32 × 10^−2^
P01008	SERPINC1	Antithrombin-III	1.9	2.66 × 10^−2^
Q96HY7	DHTKD1	Probable 2-oxoglutarate dehydrogenase E1 component	1.9	4.54 × 10^−2^
P39059	COL15A1	Collagen alpha-1(XV) chain	1.8	3.28 × 10^−2^
P35754	GLRX	Glutaredoxin-1	1.8	4.58 × 10^−2^
P40121	CAPG	Macrophage-capping protein	1.8	2.32 × 10^−2^
P36269	GGT5	Glutathione hydrolase 5 proenzyme	1.8	3.04 × 10^−2^
O75340	PDCD6	Programmed cell death protein 6	1.8	9.00 × 10^−3^
P12109	COL6A1	Collagen alpha-1(VI) chain	1.8	3.09 × 10^−2^
P19827	ITIH1	Inter-alpha-trypsin inhibitor heavy chain H1	1.7	4.77 × 10^−2^
P05109	S100A8	Protein S100-A8	1.7	3.47 × 10^−2^
P36543	ATP6V1E1	V-type proton ATPase subunit E 1	1.7	1.10 × 10^−2^
P04040	CAT	Catalase	1.7	2.98 × 10^−2^
P12110	COL6A2	Collagen alpha-2(VI) chain	1.7	3.98 × 10^−2^
P62191	PSMC1	26S proteasome regulatory subunit 4	1.7	3.55 × 10^−2^
Q16647	PTGIS	Prostacyclin synthase	1.6	2.95 × 10^−2^
Q9NZN3	EHD3	EH domain-containing protein 3	1.6	6.77 × 10^−3^
Q9UJZ1	STOML2	Stomatin-like protein 2, mitochondrial	1.6	2.81 × 10^−2^
P25786	PSMA1	Proteasome subunit alpha type-1	1.6	3.65 × 10^−2^
Q8IUE6	HIST2H2AB	Histone H2A type 2-B	1.5	1.13 × 10^−2^
Q13084	MRPL28	Mitochondrial ribosomal protein L28	1.5	5.01 × 10^−2^
Q13162	PRDX4	Peroxiredoxin-4	1.5	3.24 × 10^−2^
Q9Y490	TLN1	Talin-1	1.5	2.94 × 10^−2^
Q15121	PEA15	Astrocytic phosphoprotein PEA-15	1.5	3.16 × 10^−2^
Q9H4M9	EHD1	EH domain-containing protein 1	1.5	1.64 × 10^−2^
O75396	SEC22B	Vesicle-trafficking protein SEC22b	1.5	7.62 × 10^−3^
P24821	TNC	Tenascin	1.4	4.70 × 10^−3^
P02766	TTR	Transthyretin	1.4	4.35 × 10^−3^
P02042	HBD	Hemoglobin subunit delta	1.4	2.66 × 10^−2^
P78527	PRKDC	DNA-dependent protein kinase catalytic subunit	1.4	4.48 × 10^−2^
P25788	PSMA3	Proteasome subunit alpha type-3	1.4	4.59 × 10^−2^
P68871	HBB	Hemoglobin subunit beta	1.4	1.54 × 10^−2^
P07451	CA3	Carbonic anhydrase 3	1.4	4.69 × 10^−2^
P26447	S100A4	S100 calcium binding protein A4	1.4	9.24 × 10^−3^
P0C0S5	H2AFZ	Histone H2A.Z	1.4	1.23 × 10^−2^
P51398	DAP3	28S ribosomal protein S29, mitochondrial	1.4	4.85 × 10^−4^
P28072	PSMB6	Proteasome subunit beta type-6	1.4	3.67 × 10^−2^
P23284	PPIB	Peptidyl-prolyl cis-trans isomerase B	1.4	2.32 × 10^−2^
P04196	HRG	Histidine-rich glycoprotein	1.3	4.56 × 10^−2^
Q14697	GANAB	Neutral alpha-glucosidase AB	1.3	4.40 × 10^−2^
P00915	CA1	Carbonic anhydrase 1	1.3	1.55 × 10^−2^
Q96QK1	VPS35	Vacuolar protein sorting-associated protein 35	1.3	2.98 × 10^−2^
P69905	HBA2	Hemoglobin subunit alpha 2	1.3	3.55 × 10^−2^
P35613	BSG	Basigin	0.76	7.53 × 10^−3^
P51649	ALDH5A1	Succinate-semialdehyde dehydrogenase, mitochondrial	0.76	2.80 × 10^−2^
P62263	RPS14	Ribosomal protein S14	0.76	4.03 × 10^−2^
P09429	HMGB1	High mobility group protein B1	0.76	5.01 × 10^−2^
Q9H7Z7	PTGES2	Prostaglandin E synthase 2	0.76	3.68 × 10^−2^
Q9Y2Z9	COQ6	Ubiquinone biosynthesis monooxygenase COQ6	0.75	1.62 × 10^−2^
Q8NDY3	ADPRHL1	[Protein ADP-ribosylarginine] hydrolase-like protein 1	0.75	4.76 × 10^−2^
Q7Z406	MYH14	Myosin-14	0.75	4.69 × 10^−2^
Q16698	DECR1	2,4-dienoyl-CoA reductase, mitochondrial	0.75	4.16 × 10^−2^
Q03252	LMNB2	Lamin-B2	0.75	8.66 × 10^−3^
P10916	MYL2	Myosin regulatory light chain 2, ventricular/cardiac	0.75	2.03 × 10^−2^
Q0ZGT2	NEXN	Nexilin	0.74	4.74 × 10^−2^
E7EW31	PROB1	Proline rich basic protein 1	0.74	4.33 × 10^−2^
P52179	MYOM1	Myomesin-1	0.73	8.85 × 10^−3^
Q9Y265	RUVBL1	RuvB-like 1	0.73	3.98 × 10^−2^
P46777	RPL5	60S ribosomal protein L5	0.73	3.86 × 10^−3^
P30038	ALDH4A1	Delta-1-pyrroline-5-carboxylate dehydrogenase	0.73	2.60 × 10^−2^
P32119	PRDX2	Peroxiredoxin-2	0.73	2.77 × 10^−2^
P45379	TNNT2	Troponin T, cardiac muscle	0.73	8.78 × 10^−3^
O75521	ECI2	Enoyl-CoA delta isomerase 2, mitochondrial	0.72	4.43 × 10^−2^
Q16836	HADH	Hydroxyacyl-coenzyme A dehydrogenase, mitochondrial	0.72	1.58 × 10^−2^
Q9BTZ2	DHRS4	Dehydrogenase/reductase SDR family member 4	0.72	1.67 × 10^−2^
P07339	CTSD	Cathepsin D	0.72	1.31 × 10^−3^
Q9BX66	SORBS1	Sorbin and SH3 domain-containing protein 1	0.72	3.43 × 10^−2^
Q15046	KARS	Lysine—tRNA ligase	0.71	2.49 × 10^−2^
O75438	NDUFB1	NADH dehydrogenase [ubiquinone] 1 beta subcomplex 1	0.71	1.62 × 10^−2^
O95817	BAG3	BAG family molecular chaperone regulator 3	0.71	3.99 × 10^−2^
Q9UL25	RAB21	Ras-related protein Rab-21	0.71	3.92 × 10^−2^
P21397	MAOA	Amine oxidase [flavin-containing] A	0.70	7.04 × 10^−3^
Q96HS1	PGAM5	Serine/threonine-protein phosphatase PGAM5	0.70	1.43 × 10^−2^
Q9Y5J7	TIMM9	Mitochondrial import inner membrane translocase	0.70	1.36 × 10^−2^
O75947	ATP5H	ATP synthase subunit d, mitochondrial	0.70	2.39 × 10^−2^
P19429	TNNI3	Troponin I, cardiac muscle	0.69	9.61 × 10^−3^
P62753	RPS6	40S ribosomal protein S6	0.69	2.02 × 10^−2^
Q13045	FLII	Protein flightless-1 homolog	0.69	1.78 × 10^−2^
O95292	VAPB	Vesicle-associated membrane protein-associated protein	0.68	1.72 × 10^−2^
P02511	CRYAB	Alpha-crystallin B chain	0.67	3.99 × 10^−3^
P15121	AKR1B1	Aldose reductase	0.67	5.01 × 10^−2^
Q9NUJ1	ABHD10	Mycophenolic acid acyl-glucuronide esterase	0.67	1.49 × 10^−3^
P46779	RPL28	60S ribosomal protein L28	0.67	3.76 × 10^−2^
Q6PIU2	NCEH1	Neutral cholesterol ester hydrolase 1	0.67	4.07 × 10^−2^
Q8WWI1	LMO7	LIM domain only protein 7	0.66	5.01 × 10^−2^
Q9UFN0	NIPSNAPA	Nipsnap homolog 3A	0.66	2.13 × 10^−3^
Q14118	DAG1	Dystroglycan	0.66	7.65 × 10^−3^
Q5BKX8	MURC	Caveolae-associated protein 4	0.65	1.78 × 10^−3^
Q6IAA8	LAMTOR1	Ragulator complex protein LAMTOR1	0.65	2.14 × 10^−2^
O43676	NDUFB3	NADH dehydrogenase [ubiquinone] 1 beta subcomplex 3	0.65	3.38 × 10^−2^
P49368	CCT3	T-complex protein 1 subunit gamma	0.64	1.01 × 10^−2^
P63220	RPS21	Ribosomal protein S21	0.64	1.14 × 10^−2^
Q9UMR2	DDX19B	ATP-dependent RNA helicase DDX19B	0.64	5.87 × 10^−5^
Q8NBN7	RDH13	Retinol dehydrogenase 13	0.64	4.74 × 10^−2^
Q14192	FHL2	Four and a half LIM domains protein 2	0.64	5.01 × 10^−2^
O76024	WFS1	Wolframin	0.64	4.83 × 10^−2^
P30084	ECHS1	Enoyl-CoA hydratase, mitochondrial	0.63	5.17 × 10^−3^
Q9HBL7	PLGRKT	Plasminogen receptor (KT)	0.63	2.44 × 10^−2^
P26440	IVD	Isovaleryl-CoA dehydrogenase, mitochondrial	0.63	2.67 × 10^−2^
P60866	RPS20	Ribosomal protein S20	0.62	3.18 × 10^−2^
Q6DN03	HIST2H2BC	Putative histone H2B type 2-C	0.62	1.71 × 10^−2^
Q99460	PSMD1	26S proteasome non-ATPase regulatory subunit 1	0.61	1.71 × 10^−2^
Q96PE7	MCEE	Methylmalonyl-CoA epimerase, mitochondrial	0.61	1.44 × 10^−2^
Q8N3J5	PPM1K	Protein phosphatase 1K, mitochondrial	0.61	2.98 × 10^−2^
Q9Y5K5	UCHL5	Ubiquitin carboxyl-terminal hydrolase isozyme L5	0.61	1.82 × 10^−4^
Q9H987	SYNPO2L	Synaptopodin 2-like protein	0.60	2.82 × 10^−2^
O95182	NDUFA7	NADH dehydrogenase [ubiquinone] 1 alpha 7	0.59	8.36 × 10^−3^
Q9NQR4	NIT2	Omega-amidase NIT2	0.59	1.48 × 10^−2^
Q96ND0	FAM210A	Protein FAM210A	0.58	3.74 × 10^−2^
P46109	CRKL	Crk-like protein	0.58	2.56 × 10^−2^
Q13409	DYNC1I2	Cytoplasmic dynein 1 intermediate chain 2	0.57	9.61 × 10^−3^
P62906	RPL10A	60S ribosomal protein L10a	0.57	1.81 × 10^−2^
Q8NF91	SYNE1	Nesprin-1	0.57	2.02 × 10^−2^
O94875	SORBS2	Sorbin and SH3 domain-containing protein 2	0.56	2.55 × 10^−2^
Q9BS92	NIPSNAP3B	Nipsnap homolog 3B	0.56	5.01 × 10^−2^
Q13151	HNRNPA0	Heterogeneous nuclear ribonucleoprotein A0	0.55	1.94 × 10^−2^
P82675	MRPS5	Mitochondrial ribosomal protein S5	0.55	1.17 × 10^−2^
P22830	FECH	Ferrochelatase, mitochondrial	0.55	9.32 × 10^−3^
Q5HYJ1	TECRL	trans-2,3-enoyl-CoA reductase like	0.55	4.59 × 10^−3^
Q9Y285	FARSA	phenylalanyl-tRNA synthetase alpha subunit	0.54	2.10 × 10^−4^
Q8TB22	SPATA20	Spermatogenesis-associated protein 20	0.53	4.46 × 10^−3^
Q9NP72	RAB18	Ras-related protein Rab-18	0.52	4.87 × 10^−3^
O75190	DNAJB6	DnaJ homolog subfamily B member 6	0.52	8.53 × 10^−3^
P35542	SAA4	Serum amyloid A-4 protein	0.51	3.04 × 10^−2^
E9PAV3	NACA	Nascent polypeptide-associated complex subunit alpha	0.49	5.23 × 10^−5^
Q8N6M3	FITM2	Fat storage-inducing transmembrane protein 2	0.48	1.85 × 10^−2^
P27816	MAP4	Microtubule-associated protein 4	0.47	4.48 × 10^−2^
P0CAP1	MYZAP	Myocardial zonula adherens protein	0.47	1.87 × 10^−2^
Q96AB3	ISOC2	Isochorismatase domain containing 2	0.46	9.70 × 10^−3^
Q562R1	ACTBL2	Beta-actin-like protein 2	0.44	3.63 × 10^−2^
Q9NS69	TOMM22	Mitochondrial import receptor subunit TOM22 homolog	0.43	1.34 × 10^−2^
Q9BXK5	BCL2L13	Bcl-2-like protein 13	0.42	2.93 × 10^−2^
Q16543	CDC37	Hsp90 co-chaperone Cdc37	0.42	5.00 × 10^−2^
P43155	CRAT	Carnitine O-acetyltransferase	0.42	1.24 × 10^−3^
Q5VWP3	MLIP	Muscular LMNA-interacting protein	0.41	3.38 × 10^−2^
O60488	ACSL4	Long-chain-fatty-acid—CoA ligase 4	0.40	2.89 × 10^−4^
Q08722	CD47	Leukocyte surface antigen CD47	0.39	6.97 × 10^−3^
Q6Y288	B3GALTL	Beta-1,3-glucosyltransferase	0.38	4.29 × 10^−3^
P43487	RANBP1	Ran-specific GTPase-activating protein	0.35	1.93 × 10^−6^
P01040	CSTA	Cystatin-A	0.33	3.50 × 10^−2^
P48506	GCLC	Glutamate—cysteine ligase catalytic subunit	0.33	4.30 × 10^−2^
P08621	SNRNP70	U1 small nuclear ribonucleoprotein 70 kDa	0.30	2.09 × 10^−3^
P23919	DTYMK	Thymidylate kinase	0.30	6.33 × 10^−3^
O95394	PGM3	Phosphoacetylglucosamine mutase	0.29	4.82 × 10^−2^
P12829	MYL4	Myosin light chain 4	0.26	2.84 × 10^−4^
Q9NX40	OCIAD1	OCIA domain-containing protein 1	0.19	1.96 × 10^−3^
Q08830	FGL1	Fibrinogen-like protein 1	0.12	4.76 × 10^−3^
Q08397	LOXL1	Lysyl oxidase homolog 1	0.08	2.20 × 10^−2^

## Supplementary Materials

The following are available online at https://www.mdpi.com/1422-0067/21/18/6832/s1, Figure S1: Myocardial histology in FFPE heart samples. Table S1: Detailed information of the donors for FFPE tissue samples. Table S2: All identified and quantified human FFPE heart proteins using label-free proteomics, Table S3: All peptides identified and quantified by label free quantification approach. Table S4: Coefficient of variation (CV) of the intensity of all identified proteins in each sample. Table S5: IPA Analysis of significantly affected pathways and networks in FFPE heart proteome. Table S6. Masson’s Trichrome-stained paraffin sections and corresponding fibrotic section areas per ROI (%), as determined by digital image analysis.

## Abbreviations

MDPI	Multidisciplinary Digital Publishing Institute
DOAJ	Directory of open access journals
FFPE	Formalin-Fixed Paraffin-Embedded
IHD	ischemic heart disease
PCA	principal component analysis
pI	Isoelectric points
CV	coefficient of variation
IR	Irradiated
Ctrl	control
IPA	Ingenuity Pathway Analysis
PPAR alpha	peroxisome proliferator-activated receptor alpha
TGFB	transforming growth factor beta-1
ROI	region of interest
FASP	filter-aided sample preparation
HE	Hematoxylin and eosin-stained paraffin sections
