# Supplementary material for: Chronic Occupational Exposure to Ionizing Radiation Induces Alterations in the Structure and Metabolism of the Heart: A Proteomic Analysis of Human Formalin-Fixed Paraffin-Embedded (FFPE) Cardiac Tissue
Source: Int J Mol Sci. 2020 Sep 17;21(18):6832. doi: 10.3390/ijms21186832 (PMC7555548; doi:10.3390/ijms21186832)

**Chronic occupational exposure to ionizing radiation induces alterations in the structure and metabolism of the heart: A proteomic analysis of human formalin-fixed paraffin-embedded (FFPE) cardiac tissue**

**Omid Azimzadeh^1^, Tamara Azizova^2^, Juliane Merl-Pham^3^, Andreas Blutke^4^, Maria Moseeva^2^, Olga Zubkova^2^, Natasa Anastasov^1^, Annette Feuchtinger^4^, Stefanie M. Hauck^3^, Michael J. Atkinson^1,5^, and Soile Tapio^1^**

**Supplementary Figure S1. Myocardial histology in FFPE heart samples.** Hematoxilin-eosin stained sections of control (A) and irradiated (B). Myocytes (m) stain red, fibrous connective tissue (f) stains blue. Arrows indicate degenerating cardiomyocytes entrapped by fibrosis. Bars = 5 mm (50 µm in insets). Sections were stained with Mason’s Trichrome for visualization of fibrosis and he fibrosis area was shown in representative sections of the myocardium of control and irradiated samples (C). Area-density of fibrosis in examined sections shown in % fibrosis area/ROI (Table S5).


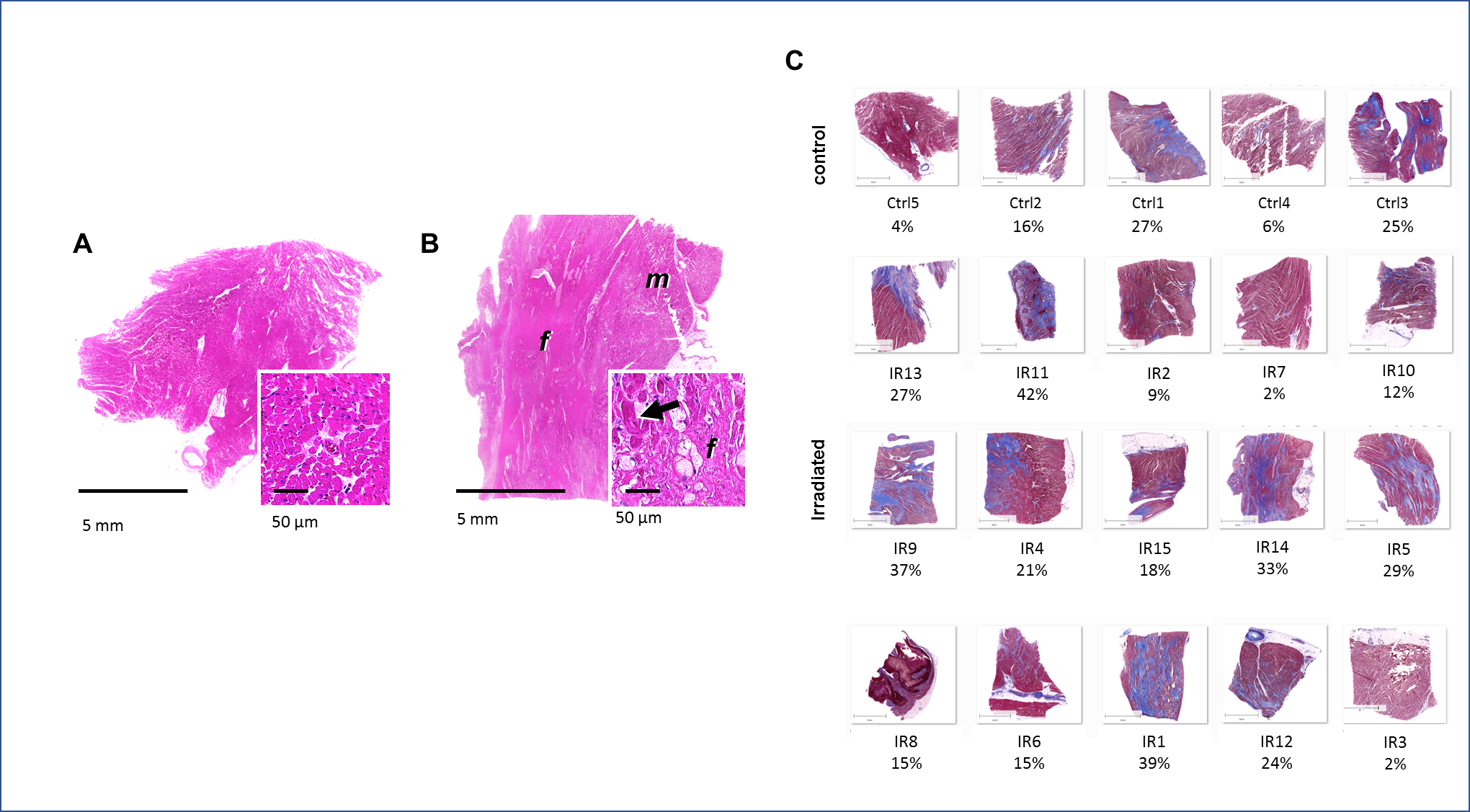

Supplement: Supplementary file 1 [file ijms-21-06832-s001.zip › ijms-856560_Supplementary Figure_18062020.docx]
